# Estimation of morphological variation in seed traits of *Sophora moorcroftiana* using digital image analysis

**DOI:** 10.3389/fpls.2023.1185393

**Published:** 2023-05-29

**Authors:** Rui Dong, Qiqiang Guo, Huie Li, Jiangrong Li, Weiwei Zuo, Cha Long

**Affiliations:** ^1^ Department of Grassland Science, College of Animal Science, Guizhou University, Guiyang, China; ^2^ Institute for Forest Resources and Environment of Guizhou, Guizhou University, Guiyang, China; ^3^ College of Agriculture, Guizhou University, Guiyang, China; ^4^ Key Lab Forest Ecology Tibet Plateau, Ministry Education, Tibet Agriculture & Animal Husbandry University, Nyingchi, China

**Keywords:** *Sophora moorcroftiana*, seed traits, genotypic variation, image analysis, digital technologies

## Abstract

*Sophora moorcroftiana* is a leguminous plant endemic to the Qinghai-Tibet Plateau. It has excellent abiotic stress tolerance and is considered an ideal species for local ecological restoration. However, the lack of genetic diversity in the seed traits of *S. moorcroftiana* hinders its conservation and utilization on the plateau. Therefore, in this study, genotypic variation and phenotypic correlations were estimated for nine seed traits among 15 accessions of *S. moorcroftiana* over two years, 2014 and 2019, respectively from 15 sample points. All traits evaluated showed significant (*P*< 0.05) genotypic variation. In 2014, accession mean repeatability was high for seed perimeter, length, width, and thickness, and 100-seed weight. In 2019, mean repeatability for seed perimeter and thickness, and 100-seed weight were high. The estimates of mean repeatability for seed traits across the two years ranged from 0.382 for seed length to 0.781 for seed thickness. Pattern analysis showed that 100-seed weight was significantly positively correlated with traits such as seed perimeter, length, width, and thickness, and identified populations with breeding pool potential. In the biplot, principal components 1 and 2 explained 55.22% and 26.72% of the total variation in seed traits, respectively. These accessions could produce breeding populations for recurrent selection to develop *S. moorcroftiana* varieties suitable for restoring the fragile ecological environment of the Qinghai-Tibet Plateau.

## 1 Introduction

The Qinghai-Tibet Plateau, with an average altitude of more than 4,000 m above sea level (m a.s.l.), is called the “Roof of the World” or the “Third-Pole on Earth” (Chen et al., 2020). The area is approximately 2.5 million km^2^, accounting for a fourth of China’s total territorial land (Dong et al., 2020; Shi et al., 2021). Owing to its characteristics of high terrain and low oxygen, the unique biological resources of the Qinghai-Tibet Plateau play a vital role in global biodiversity (Tao et al., 2020). At the same time, although different terrain and topography create a large number of diverse habitats for plants, the ecosystem in this region is fragile, and the vegetation is extremely sensitive to global climate change (Chen et al., 2020; Deng et al., 2020).

*Sophora moorcroftiana* (Benth.) Baker, is a perennial deciduous dwarf shrub of the legume family endemic to the Qinghai-Tibet Plateau. It has strong ecological adaptability, such as drought resistance, barren tolerance, and wind and sand resistance. It is mainly distributed in the valleys and hillsides of the Yarlung Zangbo River Basin at an altitude of 2,800–4,500 m above sea level (m a.s.l.) and is a dominant pioneer plant among drought-tolerant shrubs (Liu et al., 2020) and the preferred tree species for ecological restoration in the plateau. Further, low-polarity compounds contained in the seeds, such as matrine and sophocarpine, can be used to treat *Echinococcus granulosus* infections (Luo et al., 2018).

Seeds provide plants with an evolutionary advantage that allows them to survive and develop in drier places/times, store energy and nutrients to support initial development and growth, increase offspring fitness, and allow colonization and survival in adverse environments (Niklas et al., 2008; Lamont and Groom, 2013; Saatkamp et al., 2019). A range of seed morphological characteristics (e.g., seed size and epidermal characteristics) and physiological traits can coordinate the timing of seed germination under conditions suitable for seedling establishment (Saatkamp et al., 2019). In addition, both seed shape and size traits are useful for analyzing plant biodiversity and can be used to characterize intra- and inter-species variation as well as genotypic discrimination, and their correlation information is important for breeding, targeting seed yield and quality (Cervantes et al., 2016; Saatkamp et al., 2019; Khamassi et al., 2021). For example, seed mass has been identified as a key plant fitness-related trait, with larger seeds conferring advantages to plants in properties such as drought tolerance during seedling establishment, compared to small-seeded plants (Cochrane et al., 2015). This trait may reflect a trade-off for plants to develop short-term reductions in reproductive success (e.g., reduced seed production) with reduced long-term risk (Venable, 2007).

Compared to other plant organs such as flowers and leaves, using seed traits to characterize the genetic diversity of species has certain advantages because seeds are relatively easier to collect and store (Grillo et al., 2010). Pinna et al. (2014) used seed morphology parameters to analyze the interspecific, specific, and intraspecific levels of 10 *Juniperus* populations collected from the Mediterranean. Khamassi et al. (2021) characterized and evaluated the seed morphology of 24 local faba bean (*Vicia faba*) accessions and found that accessions with a white hilum were associated with lower mature grain content. Bacchetta et al. (2008) measured seed morphological characteristics for 220 accessions in the Sardinian Germplasm Bank using digital image analysis techniques and concluded that the method could be used to identify very similar taxa in these species with an accuracy of 83.7%–100%. At present, the precise quantification of seed morphological characteristics is facilitated by the development and use of digital techniques, quantification, and modeling methods (Cervantes et al., 2016).

Therefore, in this study, the variation in seed morphological characteristics of 15 *S. moorcroftiana* populations collected from different locations on the Qinghai-Tibet Plateau was studied using digital image analysis techniques. Our research focused on estimating the genetic variation within and among populations. In addition, a combination of potentially beneficial seed traits has been evaluated in breeding programs. This study aimed to provide data support for the genetic diversity and taxonomy of *S. moorcroftiana*, and to provide valuable parameters and information for the selection and breeding of strong adaptability *S. moorcroftiana* varieties.

## 2 Materials and methods

### 2.1 Germplasm

**Figure 1:**
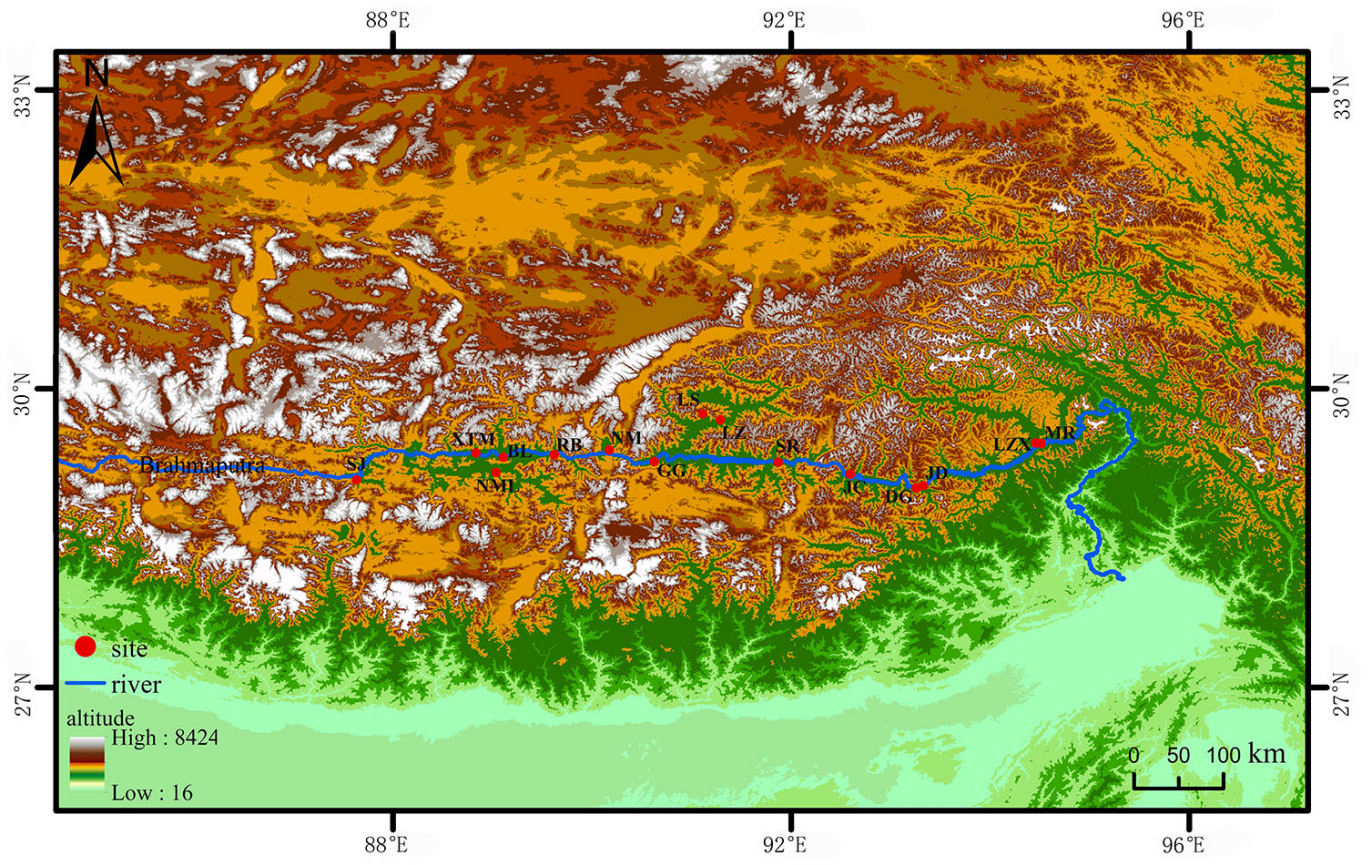
The distribution of 15 *S. moorcroftiana* accessions used in this study. The red dots represent the sampling points. LZX, Lin zhi; NML, Nan mu lin; GG, Gong ga; RB, Ren bu; JC, Jia cha; LS, La sa; JD, Jin dong; BL, Bai lang; XTM, Xie tong men; DG, Dong ga; SJ, Sa jia; NM, Ni mu; SR, Sang ri; MR, Mi rui; LZ, Lin zhou.

The seed resources of 15 accessions were evaluated in this study. *S. moorcroftiana* seeds were collected at 15 sampling points during October 1–7, 2014, and October 1–7, 2019 (**Figure 1**). The collected seeds were dried to a moisture content of 6%–8% and stored at 4°C and 30%–50% relative humidity. The climate data of the sampling points are provided by the meteorological data center of the China meteorological administration.

### 2.2 Trait measurements

Nine seed traits were measured: SL, seed length (mm); SW, seed width (mm); W/L, seed width to seed length ratio; HL, hilum length (mm); HW, hilum width (mm); HW/HL, hilum width to hilum length ratio; Pe, perimeter (mm); ST, seed thickness (mm) and SY, 100-seed weight (g). 100 seeds were manually counted. Use an electronic balance (Sartorius, BSA224S-CW, China) for weighing. Before trait measurements, a flatbed scanner (EPSON GT-15000) was used to obtain digital images of the seed samples. During the scan, the seeds were allowed to equilibrate before measurement (room temperature was maintained at 20 ± 3°C and 40 ± 5% relative humidity) (Grillo et al., 2010). The scanned image resolution was 200 dpi, and the number of pixels was 1024 × 1024.

Three replicates were scanned for each population and each replicate included 100 seeds. Seed samples were prepared and scanned according to methods described by Venora et al. (2007); Bacchetta et al. (2008), and Dong et al. (2016). A WinSEEDLE 2011 image analysis system was used to process the acquired images (Dong et al., 2016).

### 2.3 Data analysis

Data analysis was based on (1) variance component analysis to assess the magnitude and significance of genotypic variation between populations and (2) pattern analysis, including a combination of clustering and principal component analysis (PCA) (Dong et al., 2016; Dong et al., 2019) to provide a graphical summary of the multi-trait data matrices.

Data on seed traits from 15 *S. moorcroftiana* population accessions were analyzed within and over two years (2014 and 2019). The analyses were conducted using the variance component analysis procedure, residual maximum likelihood (REML), in GenStat 7.1 (2003) (Dong et al., 2019). Analysis of data over the years was performed using a mixed linear model (Dong et al., 2019).

All seed trait means were derived from the best linear unbiased predictor (BLUP) analysis (White and Hodge, 1989; Dong et al., 2019). These BLUP values were used to build a population × trait mean matrix adjusted for population × year interaction effects.

Referring to Fehr (1987) method, the estimated genotypic ( $\sigma_{\;g}^{2}$ ), genotype × year interaction ( $\sigma_{\;g}^{2}$ ), experimental error ( $\sigma_{\;\varepsilon }^{2}$ ), *nl* (number of years), and *nr* (number of replications) obtained from REML analysis were used to estimate the population accession mean repeatability (*R*).

Accession mean repeatability within a single year:


(1) $$R_{1}=\frac{\sigma_{g}^{\;2}}{\sigma_{g}^{\;2}+\frac{\sigma_{\varepsilon }^{\;2}}{n_{r}}}$$

Accession mean repeatability across years:


(2) $$R_{2}=\frac{\sigma_{g}^{\;2}}{\sigma_{g}^{\;2}+\frac{\sigma_{gl}^{\;2}}{n_{l}}+\frac{\sigma_{\varepsilon }^{\;2}}{n_{l}n_{r}}}$$

Phenotypic correlation (*rp*) analysis was performed using GenStat 7.1 (2003), and multivariate analysis of variance (MANOVA) was used to assess accessions for 15 populations over two years, resulting in the sum of the estimated cross-products of the multi-trait data matrix.

Pattern analysis is a combination of cluster analysis and principal component analysis (PCA): a) based on the variance components over the two years 2014 and 2019 to obtain an adjusted mean matrix of genotype × trait BLUP and finally obtain a graphical summary of accession traits for eight populations; and b) to analyze the type of association (positive or negative) among the nine seed traits in 2014 and 2019.

## 3 Results

### 3.1 Genotypic variance components and the mean repeatability of nine seed traits of *S. moorcroftiana*

The genotypic variance components of the nine seed traits in 2014 and 2019 showed significant differences (*P*< 0.05) for all the traits evaluated in the 15 *S. moorcroftiana* accessions (**Tables 1**, **2**).

**Table 1:** Average, maximum, minimum, least significant differences (*l.s.d._0.05_*), estimated genotypic ( $\sigma_{\;g}^{2}$ ) and experimental error ( $\sigma_{\;\varepsilon }^{2}$ ) variance components and associated standard errors ( ± SE), and mean repeatability (*R_1_*) estimated from the 15 *S. moorcroftiana* accessions, evaluated in 2014.

Traits	Perimeter	Seed Length	Seed Width	Seed width/Seed Length	Hilum length	Hilum width	Hilum width/Hilum length	Seed thickness	100-seed weight
Average	14.661	4.453	4.405	0.990	1.790	1.295	0.725	3.594	0.791
Min	13.626	4.157	4.066	0.951	1.612	1.171	0.683	3.305	0.553
Max	15.262	4.644	4.611	1.023	1.918	1.516	0.797	3.711	0.900
*l.s.d._0.05_*	0.491*	0.157*	0.143*	0.044*	0.117*	0.093*	0.040*	0.107*	0.064*
*σ* ^2^ *_g_*	0.211 ± 0.091	0.016 ± 0.007	0.016 ± 0.007	0.001 ± 0.001	0.004 ± 0.002	0.133 ± 0.359	0.141 ± 0.097	0.010 ± 0.004	0.009 ± 0.004
$\sigma_{\;\varepsilon }^{2}$	0.086 ± 0.023	0.007 ± 0.002	0.008 ± 0.002	0.007 ± 0.002	0.005 ± 0.001	0.152 ± 0.549	0.186 ± 0.016	0.004 ± 0.001	0.001 ± 0.003
*R_1_*	0.948	0.939	0.933	0.058	0.688	0.697	0.633	0.949	0.992

* indicates significance at P< 0.05.

**Figure 2:**
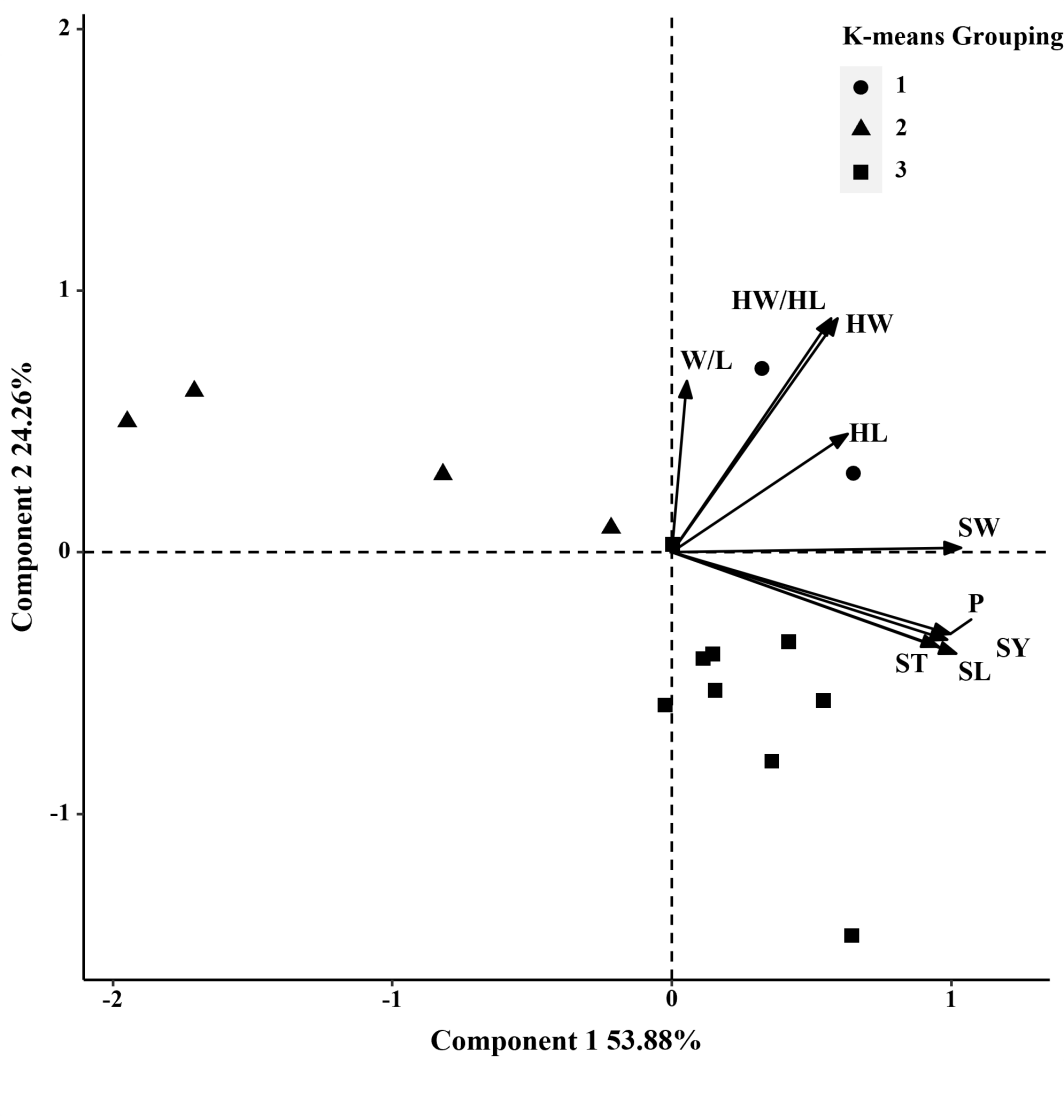
Biplot generated using standardized Best Linear Unbiased Predictor values for nine seed traits measured from 15 *S. moorcroftiana* accessions, evaluated in 2014. Components I and II account for 53.88% and 24.26% of total variation, respectively. The different symbols indicate progeny Groups 1 to 3 generated from cluster analysis.

In 2014, accession mean repeatability (*R1*) was high for seed perimeter, length, width, and thickness, and 100-seed weight, ranging from 0.933 to 0.992 (**Table 1**; **Supplementary Figure 1A**). The *R1* values for the hilum length, width, and hilum length/width ratio were intermediate, ranging from 0.633 to 0.697. *R1* for seed width/seed length was the lowest at 0.058. In 2019, *R1* for seed perimeter and thickness, and 100-seed weight were high, ranging from 0.846 to 0.991 (**Table 2**; **Supplementary Figure 1B**). *R1* for seed length, hilum length and width, and hilum length/width ratio were intermediate, ranging from 0.604 to 0.767. *R1* values for seed width and seed width/length ratio were lower than those of the other traits (0.489 and 0.054, respectively).

**Table 2:** Average, maximum, minimum, least significant differences (*l.s.d._0.05_*), estimated genotypic ( $\sigma_{\;g}^{2}$ ) and experimental error ( $\sigma_{\;\varepsilon }^{2}$ ) variance components and associated standard errors ( ± SE), and mean repeatability (*R_1_*) estimated from the 15 *S. moorcroftiana* accessions, evaluated in 2019.

Traits	Perimeter	Seed Length	Seed Width	Seed width/Seed Length	Hilum length	Hilum width	Hilum width/Hilum length	Seed thickness	100-seed weight
Average	14.959	4.592	4.554	0.992	1.773	1.307	0.738	3.730	0.876
Min	14.427	4.453	4.379	0.978	1.695	1.242	0.704	3.561	0.548
Max	15.340	4.706	4.720	1.010	1.856	1.368	0.806	3.846	0.995
*l.s.d._0.05_*	0.851*	0.263*	0.214*	0.034*	0.110*	0.070*	0.045*	0.113*	0.094*
*σ* ^2^ *_g_*	0.327 ± 0.068	0.018 ± 0.006	0.009 ± 0.008	0.008 ± 0.001	0.002 ± 0.002	0.001 ± 0.001	0.007 ± 0.001	0.007 ± 0.005	0.020 ± 0.011
$\sigma_{\;\varepsilon }^{2}$	0.242 ± 0.092	0.023 ± 0.009	0.017 ± 0.006	0.059 ± 0.001	0.003 ± 0.001	0.002 ± 0.001	0.007 ± 0.002	0.004 ± 0.002	0.003 ± 0.001
*R_1_*	0.846	0.648	0.489	0.054	0.604	0.715	0.767	0.890	0.991

* indicates significance at P< 0.05.

Analysis of variance for over two years, 2014 and 2019, showed significant genotypic variation (*P*< 0.05) among the 15 *S. moorcroftiana* accessions for nine seed traits (**Table 3**). The mean repeatability (*R2*) of hilum length/width and seed thickness was higher than that of the other traits (0.757 and 0.781, respectively). The *R2* values for the hilum length and width, and 100-seed weight were intermediate at 0.597, 0.643, and 0.626, respectively. *R2* for seed perimeter, length, width, and width/length ratio were lower than those for the other traits, ranging from 0.097 to 0.493.

**Table 3:** Average, maximum, minimum, least significant differences (*l.s.d._0.05_*), estimated genotypic ( $\sigma_{\;g}^{2}$ ), genotype × year interaction ( $\sigma_{\;gy}^{2}$ ) and experimental error ( $\sigma_{\;\varepsilon }^{2}$ ) variance components and associated standard errors ( ± SE), and mean repeatability (*R_2_*) estimated from the 15 *S. moorcroftiana* accessions, evaluated across two years, 2014 and 2019.

Traits	Perimeter	Seed Length	Seed Width	Seed width/Seed Length	Hilum length	Hilum width	Hilum width/Hilum length	Seed thickness	100-seed weight
Average	14.778	4.522	4.470	0.989	1.782	1.297	0.729	3.652	0.830
Min	13.626	4.157	4.066	0.951	1.612	1.171	0.683	3.305	0.548
Max	15.340	4.706	4.720	1.023	1.918	1.516	0.806	3.846	0.995
*l.s.d._0.05_*	0.551*	0.182*	0.181*	0.025*	0.108*	0.083*	0.035*	0.136*	0.134*
*σ* ^2^ *_g_*	0.055 ± 0.017	0.043 ± 0.006	0.072 ± 0.005	0.068 ± 0.000	0.180 ± 0.002	0.260 ± 0.221	0.016 ± 0.062	0.058 ± 0.001	0.099 ± 0.006
$\sigma_{\;\varepsilon }^{2}$	0.154 ± 0.054	0.016 ± 0.006	0.111 ± 0.004	0.042 ± 0.001	0.080 ± 0.003	0.450 ± 0.142	0.382 ± 0.135	0.004 ± 0.001	0.003 ± 0.001
*σ* ^2^ *_gy_*	0.005 ± 0.001	0.005 ± 0.002	0.008 ± 0.003	0.046 ± 0.001	0.052 ± 0.004	0.069 ± 0.030	0.003 ± 0.080	0.004 ± 0.003	0.015 ± 0.009
*R_2_*	0.441	0.382	0.493	0.097	0.597	0.643	0.757	0.781	0.626

* indicates significance at P< 0.05.

### 3.2 Pattern analysis and phenotypic correlation of *S. moorcroftiana*

In 2014, based on phenotypic correlation analysis, there was a positive correlation between 100-seed weight and seed perimeter, length, width, and thickness (**Table 4**). Seed perimeter, length, and width also exhibited strong positive correlations at the phenotypic level. In the biplot, principal components 1 and 2 explained 53.88% and 24.26% of the total variation in seed traits, respectively (**Figure 2**). The BLUP mean matrix of nine seed traits was used for the cluster analysis grouping of 15 *S. moorcroftiana* accessions in 2014, truncated at the group three level. According to the trait means for each group, Group 1 had the highest mean seed perimeter, length, width, and width/length ratio, hilum length, width, and length/width ratio, and 100-seed weight and included two accessions (**Supplementary Table 1**).

**Table 4:** Phenotypic (*r_p_*) correlation coefficients, between traits based on the 15 *S. moorcroftiana* accessions, evaluated in 2014.

Traits	Perimeter	Seed Length	Seed Width	Seed width/Seed Length	Hilum length	Hilum width	Hilum width/Hilum length	Seed thickness
Seed Length	0.947^**^							
Seed Width	0.846^**^	0.825^**^						
Seed width/Seed Length	-0.220	-0.346^*^	0.245					
Hilum length	0.384^**^	0.388^**^	0.382^**^	-0.033				
Hilum width	0.017	-0.010	0.014	0.038	0.133			
Hilum width/Hilum length	-0.006	-0.033	-0.010	0.040	0.072	0.998^**^		
Seed thickness	0.680^**^	0.725^**^	0.755^**^	0.009	0.288^*^	-0.176	-0.195	
100-seed weigh	0.668^**^	0.728^**^	0.764^**^	0.024	0.424^**^	-0.011	-0.038	0.833^**^

*, ** indicates significant at P< 0.05 and P< 0.01 levels, respectively.

**Figure 3:**
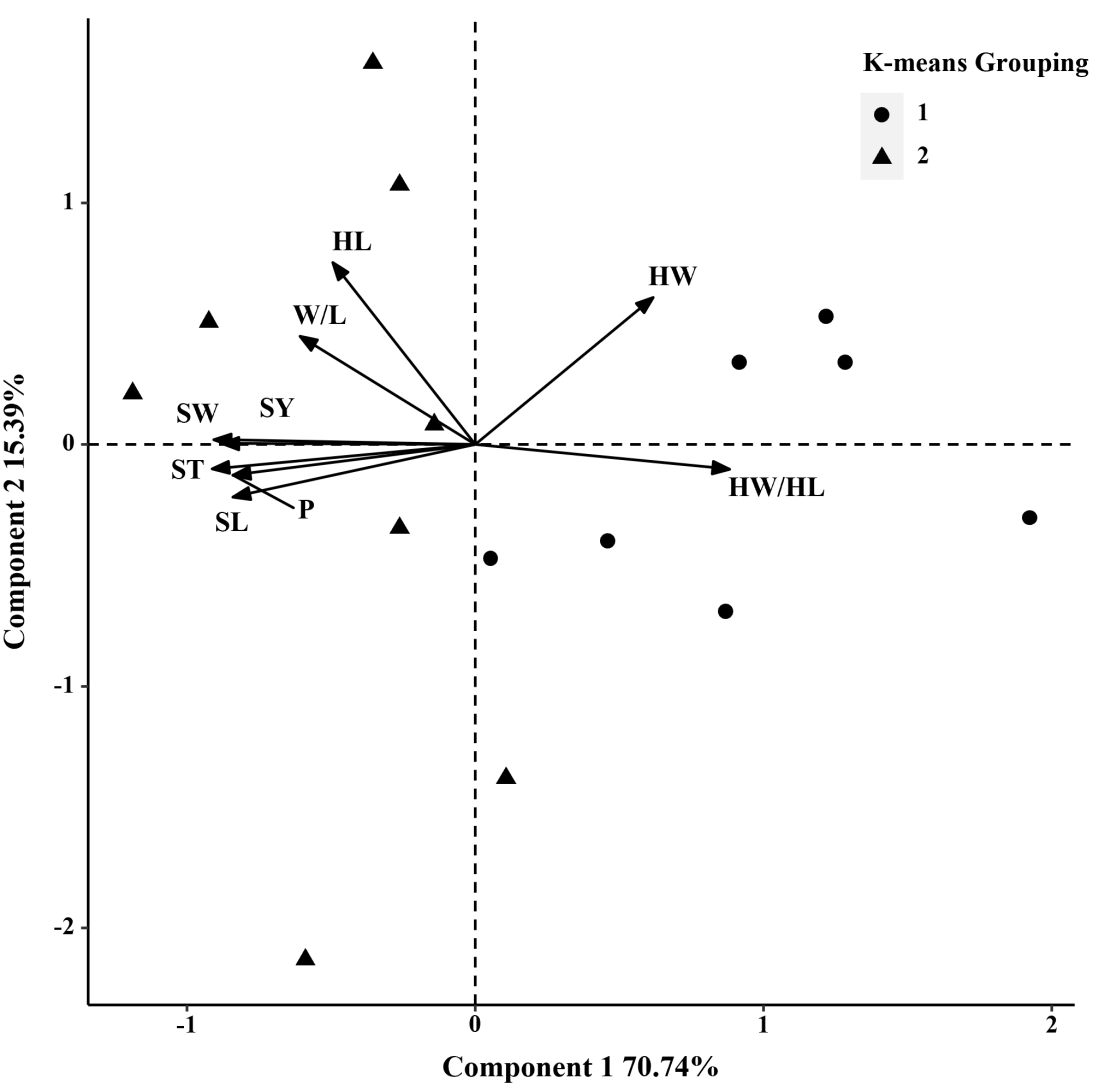
Biplot generated using standardized Best Linear Unbiased Predictor values for nine seed traits measured from 15 *S. moorcroftiana* accessions, evaluated in 2019. Components I and II account for 70.74% and 15.39% of total variation, respectively. The different symbols indicate progeny Groups 1 and 2 generated from cluster analysis.

In 2019, the phenotypic correlation analysis showed that 100-seed weight, seed perimeter, length, width, and thickness showed strong positive correlations at the phenotypic level (**Table 5**). In the biplot, principal component 1 explained 70.74% of the total seed trait variation and principal component two explained 15.39% (**Figure 3**). The 15 *S. moorcroftiana* accession groups generated from the cluster analysis were truncated at the two-group level. The results showed that the second group had a higher seed perimeter, length, width, thickness and width/length ratio, hilum length, and 100-seed weight (**Supplementary Table 2**).

**Table 5:** Phenotypic (*r_p_*) correlation coefficients, between traits based on the 15 *S. moorcroftiana* accessions, evaluated in 2019.

Traits	Perimeter	Seed Length	Seed Width	Seed width/Seed Length	Hilum length	Hilum width	Hilum width/Hilum length	Seed thickness
Seed Length	0.865^**^							
Seed Width	0.811^**^	0.658^**^						
Seed width/Seed Length	-0.068	-0.419^**^	0.408^**^					
Hilum length	0.227	0.266	0.301^*^	0.040				
Hilum width	0.014	0.025	0.131	0.126	0.252			
Hilum width/Hilum length	0.005	0.014	0.117	0.122	0.205	0.998^**^		
Seed thickness	0.622^**^	0.641^**^	0.636^**^	-0.006	0.377^*^	0.012	-0.012	
100-seed weigh	0.710^**^	0.793^**^	0.747^**^	-0.058	0.313^*^	0.062	0.047	0.762^**^

*, ** indicates significant at P< 0.05 and P< 0.01 levels, respectively.

**Figure 4:**
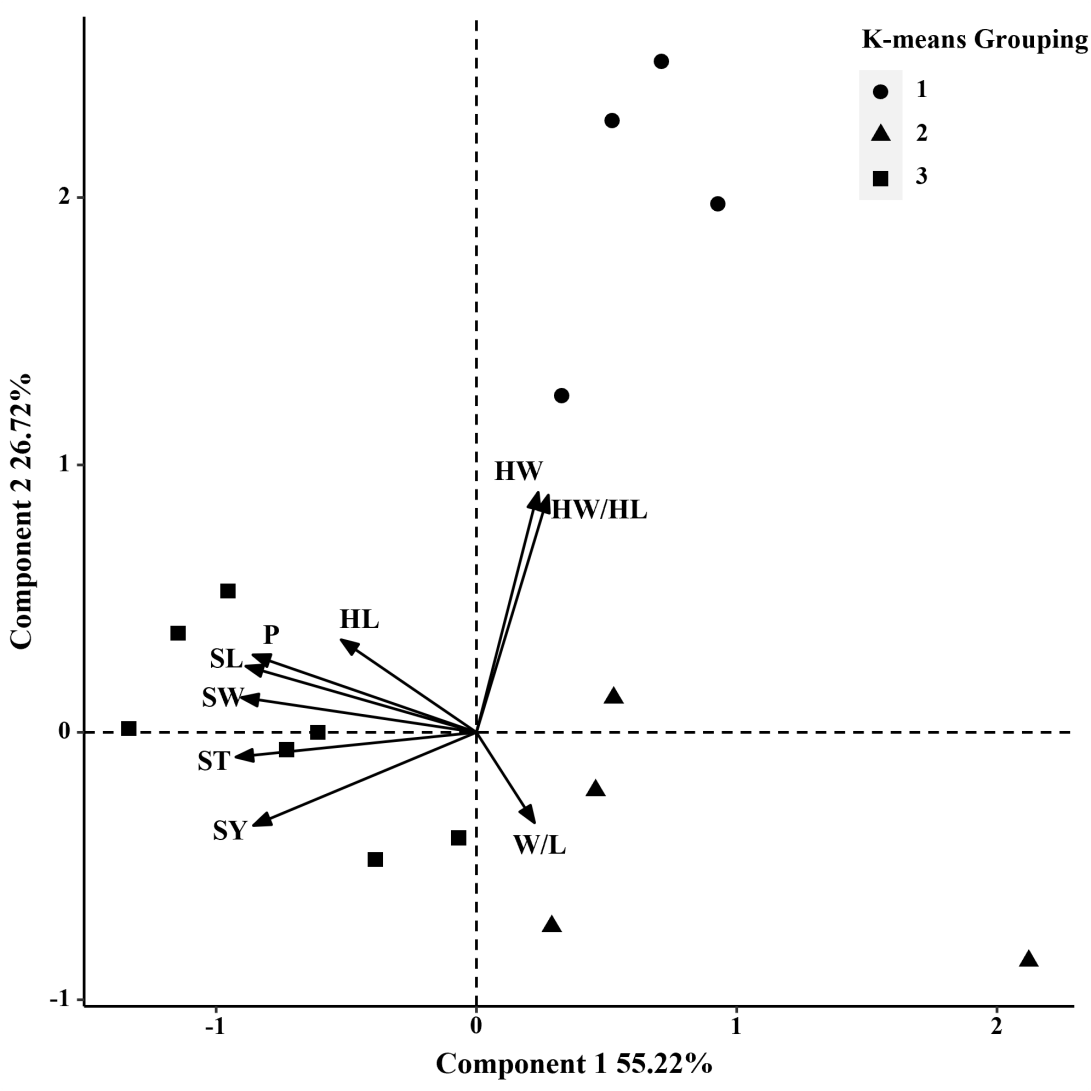
Biplot generated using standardized Best Linear Unbiased Predictor values for nine seed traits measured from 15 *S. moorcroftiana* accessions, evaluated across two years, 2014 and 2019. Components I and II account for 55.22% and 26.72% of total variation, respectively. The different symbols indicate progeny Groups 1 to 3 generated from cluster analysis.

In 2014 and 2019, based on the phenotypic correlation analysis, 100-seed weight showed a strong positive correlation with seed perimeter, length, width, and thickness at the phenotypic level and a strong negative correlation with hilum length/width ratio (**Table 6**). In the biplot, principal components 1 and 2 explained 55.22% and 26.72% of the total variation in seed traits, respectively (**Figure 4**). The 15 *S. moorcroftiana* accession groups generated from the cluster analysis were truncated at the three-group level. The third group of accessions had higher seed perimeter, seed length, width, width/length ratio, thickness, and 100-seed weight, including five accessions (**Supplementary Table 3**). Furthermore, in 2014 and 2019, the seed traits perimeter, hilum length/width ratio, seed thickness, and 100-seed weight were all significantly correlated with altitude, and hilum length/width ratio, seed thickness, and 100-seed were significantly correlated with the monthly average maximum temperature, monthly average minimum temperature, and monthly average temperature during the growing season (**Supplementary Figure 2**).

**Table 6:** Phenotypic (*r_p_*) correlation coefficients, between traits based on the 15 *S. moorcroftiana* accessions, evaluated across two years, 2014 and 2019.

Traits	Perimeter	Seed Length	Seed Width	Seed width/Seed Length	Hilum length	Hilum width	Hilum width/Hilum length	Seed thickness
Seed Length	0.954^**^							
Seed Width	0.907^**^	0.834^**^						
Seed width/Seed Length	-0.081	-0.286	0.289					
Hilum length	0.466^*^	0.434^*^	0.467^*^	0.058				
Hilum width	-0.053	-0.079	-0.215	-0.242	0.299			
Hilum width/Hilum length	-0.443^*^	-0.436^*^	-0.582^**^	-0.259	-0.610^**^	0.572^**^		
Seed thickness	0.599^**^	0.584^**^	0.755^**^	0.303	0.312	-0.554^**^	-0.735^**^	
100-seed weigh	0.562^**^	0.569^**^	0.717^**^	0.266	0.452^*^	-0.520^**^	-0.832^**^	0.859^**^

*, ** indicates significant at P< 0.05 and P< 0.01 levels, respectively.

## 4 Discussion

Numerous studies have shown that structural diversity in seed traits helps characterize both intra- and inter-species variation. Therefore, the study of macro- and micro-seed traits is important in quantifying genetic diversity and plant taxonomy (Barthlott, 1981; Grillo et al., 2010).

Previous studies on *S. moorcroftiana* have mainly focused on its population distribution (Liu et al., 2020; Xin et al., 2021; Yang et al., 2021), soil seed banks (Zhao et al., 2007), medicinal functions (Wang et al., 2014; Luo et al., 2018), analysis of transcriptome (Li et al., 2015) and verification of gene functions (Li et al., 2017). In this study, we report, for the first time, the phenotypic and genotypic variations in nine seed traits and the mean repeatability of 15 accessions of *S. moorcroftiana*.

In nature, plants growing in various environments have evolved adaptive traits related to seed morphology and physiology to cope with adverse environments, such as variability in seed size, seed dormancy characteristics, and a special structure that maintains the reproduction and spread of the population (Venable and Brown, 1988; Luzuriaga et al., 2006). These seed traits are mainly determined by the seed genotype and parental environment (Schmitt et al., 1992). At the same time, parental effects also include the result of the interaction of genotype and maternal environment. The influence of parents on offspring is partly determined by genes; therefore, they are evolvable (Lacey, 1998). In the present study, seed perimeter, seed thickness, and 100-seed weight all had high *R1* values in a single year, whereas hilum length/hilum width and seed thickness had high *R2* values across years. The relatively high genotypic variation in these traits indicated potential genetic variation among the 15 *S. moorcroftiana* accessions that could be used for selection and breeding (Dong et al., 2019). Furthermore, these seed trait variation reflects the result of genetic variation and phenotypic plasticity in response to environmental variation (Wang et al., 2023). This information helps to understand the response mechanism and variation rules of plants to the environment, which is important for the collection, preservation and evaluation of germplasm resources.

The size and weight of seeds produced by different plant species vary widely. A previous study found that seed size showed different characteristics during the growth and development of plant offspring (Moles et al., 2005). Small-seeded plant species can produce more seeds than large-seeded plant species for a given amount of energy. However, seedlings of large-seeded plant species are more resilient to biotic and abiotic stresses during their establishment. Small-seeded plant species adopt another strategy for winning by quantity: producing as many offspring as possible to ensure their survival. This suggests that traits such as seed size and weight of different species grown in a specific environment can have a major impact on seedling establishment and survival (Dong et al., 2016). Therefore, information on the genetic variation in key seed-size traits in breeding materials will facilitate the execution of various developmental programs (Odiaka, 2005). In this study, the ranges of seed perimeter, seed thickness, and 100-seed weight reflecting seed size and quality were 13.626–15.262 mm, 3.305–3.711 mm, and 0.553–0.900 g in 2014 and 14.427–15.340 mm, 3.561–3.846 mm, and 0.548–0.995 g in 2019, respectively; the *R1* of these traits was higher than 0.8, and the *R2* of seed thickness and 100-seed weight were higher than 0.6. This suggests that these traits are mainly affected by the genotype and can provide valuable information for *S. moorcroftiana* breeding. In addition, seed thickness, length, width, and perimeter, and 100-seed weight showed extremely significant positive correlations between the two years, indicating that changes in any trait may significantly affect seed weight. This result indicated that these traits were mainly determined by seed genotype and that changes in either trait could significantly affect seed weight. This positive correlation has important commercial and practical implications for breeding programs (Amiri et al., 2010; Dong et al., 2019). At the same time, variability in seed size affects seed dispersal in a variety of ways. Because smaller seeds are usually dispersed further by abiotic factors such as water and wind. This is closely related to the external environment such as the altitude, slope, temperature and rainfall of the population (Liao et al., 2020; Yang et al., 2021). This was also supported by the correlations between seed traits and altitude and temperature in this study. This phenomenon has important implications for individual reproductive success, community structure, and biodiversity patterns of plants (Snell et al., 2019).

In previous studies, pattern analysis has been successfully used to analyze nine environmental and nine genotype trait data matrices (Jahufer et al., 1997; Zhang et al., 2006; Luo et al., 2016). Jahufer et al. (1997) used pattern analysis to analyze 439 white clover germplasm resources and screened out germplasm populations characterized by large leaves, tall plants, and thick stolons, which could be used to develop varieties that can tolerate summer drought stress environments. Dong et al. (2019) analyzed the genotypic and phenotypic variation of 18 traits of 418 common vetch germplasms based on pattern analysis and obtained germplasm populations with low shattering rates, high seed yields, and high plant dry weights, which can be used for common vetch breeding programs with high seed yield and high dry plant weight. Similarly, in this study, we obtained germplasm populations with higher seed sizes and 100-seed weights using pattern analysis. These accessions could be used in *S. moorcroftiana* breeding programs with high seedling establishment success rates to adapt to the harsh natural conditions of the Qinghai-Tibet Plateau.

Seed size, shape, and epidermal surface characteristics of plants play important roles in plant morphological diversity, and these seed morphological characteristics can provide data for taxa at different taxonomic levels (Ocampo et al., 2014). In addition, the seed characteristics of plants are different from their floral features, which are generally considered to be more conserved and thus can provide valuable information on the evolutionary history of flowering plants (Barthlott, 1981). Becquer et al. (2014) studied seed shape and size, raphe shape and size, and seed coat surface morphology data of 47 Compositae species from the Antilles, providing information for phylogenetic reconstruction and trait evolution analysis. In this study, the genotypic variation in different seed traits of each accession was significantly different (*P* < 0.05), which may help to investigate their taxonomic relationships. Analysis of the seed morphological characteristics of *S. moorcroftiana* showed that the JC, JD, SR, and BL accessions were significantly different (*P* < 0.05) from the GG, RB, NML, XTM, NM, DG, and SJ accessions, which could be divided into two groups. Our study shows that these heterogeneous seed traits can provide valuable information on the evolutionary relationship of *S. moorcroftiana*, and the seed morphology database has the potential for taxonomic screening (Dell'Aquila, 2007).

Nondestructive studies based on the plant seed characterization have proven to be an informative, noninvasive, and suitable tool for differentiating germplasm resources (Sinkovič et al., 2019). The results obtained in this study are serving as the useful information on genetic diversity, plant classification and breeding of *S. moorcroftiana* accessions, which could be used for future research on the evolution, classification and population restoration of *S. moorcroftiana*.

## 5 Conclusion

This study estimated the phenotypic correlation, genotypic variation, and mean repeatability of nine seed traits in 15 *S. moorcroftiana* accessions. Seed perimeter, seed thickness, and 100-seed weight showed high mean repeatability over two years (2014 and 2019), indicating their potential for genetic improvement. Pattern analysis showed that the 100-seed weight was significantly and positively correlated with seed perimeter, length, width, and thickness. The significant correlation between these traits provides key information for *S. moorcroftiana* breeding programs that focus on developing varieties with high seedling establishment success rates. This study not only deepens our understanding of the genetic diversity of *S. moorcroftiana* seed morphological traits but also provides important information for the development of breeding banks.

## Data availability statement

The original contributions presented in the study are included in the article/**Supplementary Material**. Further inquiries can be directed to the corresponding author.

## Author contributions

RD and HL conceived the experiment. QG and JL collected the seeds. WZ and CL carried out the experiment. RD and QG analyzed the data. RD and HL wrote the paper. All the authors contributed to the manuscript and approved the submitted version. All authors contributed to the article and approved the submitted version.
