# Supplementary material for: Estimation of morphological variation in seed traits of Sophora moorcroftiana using digital image analysis
Source: Front Plant Sci. 2023 May 29;14:1185393. doi: 10.3389/fpls.2023.1185393 (PMC10258342; doi:10.3389/fpls.2023.1185393)
Supplement: Supplementary file 1 [file DataSheet_1.docx]

Supplementary Material

Estimation of morphological variation in seed traits of *Sophora moorcroftiana* using digital image analysis

Zhou Li ^1^, Qiqiang Guo^2^, Huie Li^3*^, Jiangrong Li^4^, Weiwei Zuo^3^, Cha Long^3^

^1^ Department of Grassland Science, College of Animal Science, Guizhou University, Guiyang, China

^2^ Institute for Forest Resources and Environment of Guizhou, Guizhou University, Guiyang, China

^3^ College of Agriculture, Guizhou University, Guiyang, China

^4^ Key Lab Forest Ecology Tibet Plateau, Ministry Education, Tibet Agriculture & Animal Husbandry University, Nyingchi, China

*** Correspondence:**Huie Li
lihuiesh@126.com

# Supplementary Tables

**Supplementary Table 1.** Trait means for each of the three groups of 15 *S. moorcroftiana* accessions, evaluated in 2014, generated from pattern analysis. LZX, Lin zhi; NML, Nan mu lin; GG, Gong ga; RB, Ren bu; JC, Jia cha; LS, La sa; JD, Jin dong; BL, Bai lang; XTM, Xie tong men; DG, Dong ga; SJ, Sa jia; NM, Ni mu; SR, Sang ri; MR, Mi rui; LZ, Lin zhou.

**Supplementary Table 2.** Trait means for each of the two groups of 15 *S. moorcroftiana* accessions, evaluated in 2019, generated from pattern analysis. LZX, Lin zhi; NML, Nan mu lin; GG, Gong ga; RB, Ren bu; JC, Jia cha; LS, La sa; JD, Jin dong; BL, Bai lang; XTM, Xie tong men; DG, Dong ga; SJ, Sa jia; NM, Ni mu; SR, Sang ri; MR, Mi rui; LZ, Lin zhou.

**Supplementary Table 3.** Trait means for each of the three groups of 15 *S. moorcroftiana* accessions, evaluated across two years, 2014 and 2019, generated from pattern analysis. LZX, Lin zhi; NML, Nan mu lin; GG, Gong ga; RB, Ren bu; JC, Jia cha; LS, La sa; JD, Jin dong; BL, Bai lang; XTM, Xie tong men; DG, Dong ga; SJ, Sa jia; NM, Ni mu; SR, Sang ri; MR, Mi rui; LZ, Lin zhou.

# Supplementary Figures

**Supplementary Figure 1.** Boxplots of mean values for 15 *S. moorcroftiana* accessions seed traits evaluated in 2014 and 2019. A, seed trait for 2014. B, seed trait for 2019.

**Supplementary Figure 2.** Correlations between seed traits and environments of 15 *S. moorcroftiana* accessions evaluated in 2014 and 2019. A, correlations for 2014. B, correlations for 2019. SL, seed length (mm); SW, seed width (mm); W/L, seed width to seed length ratio; HL, hilum length (mm); HW, hilum width (mm); HW/HL, hilum width to hilum length ratio; Pe, perimeter (mm); ST, seed thickness (mm); SY, 100-seed weight (g); a.s.l., above sea level (m); T max, Monthly average maximum temperature; T min, monthly average minimum temperature; T, monthly average temperature.
