# Supplementary figures and images for: Estimation of morphological variation in seed traits of Sophora moorcroftiana using digital image analysis
Source: Front Plant Sci. 2023 May 29;14:1185393. doi: 10.3389/fpls.2023.1185393 (PMC10258342; doi:10.3389/fpls.2023.1185393)

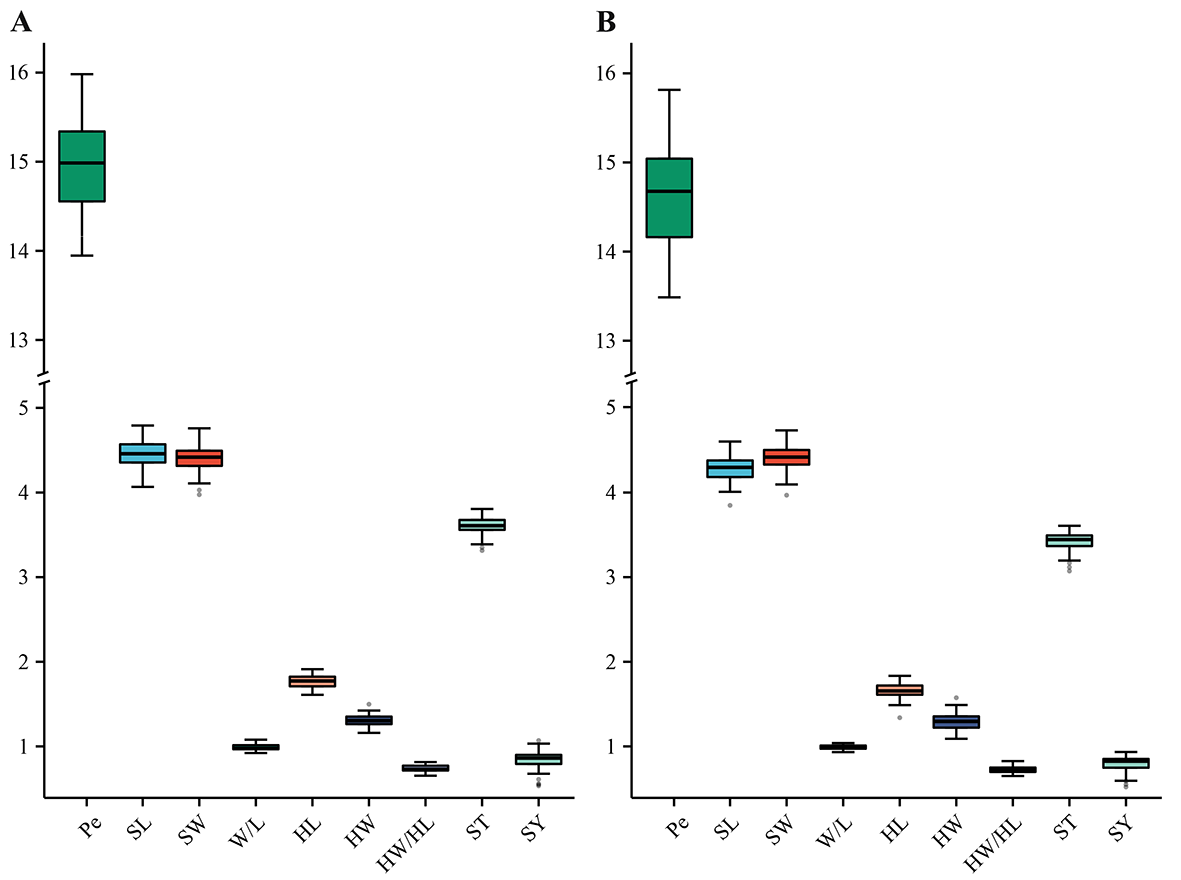

Supplement: Supplementary file 2 [file Image_1.tif]

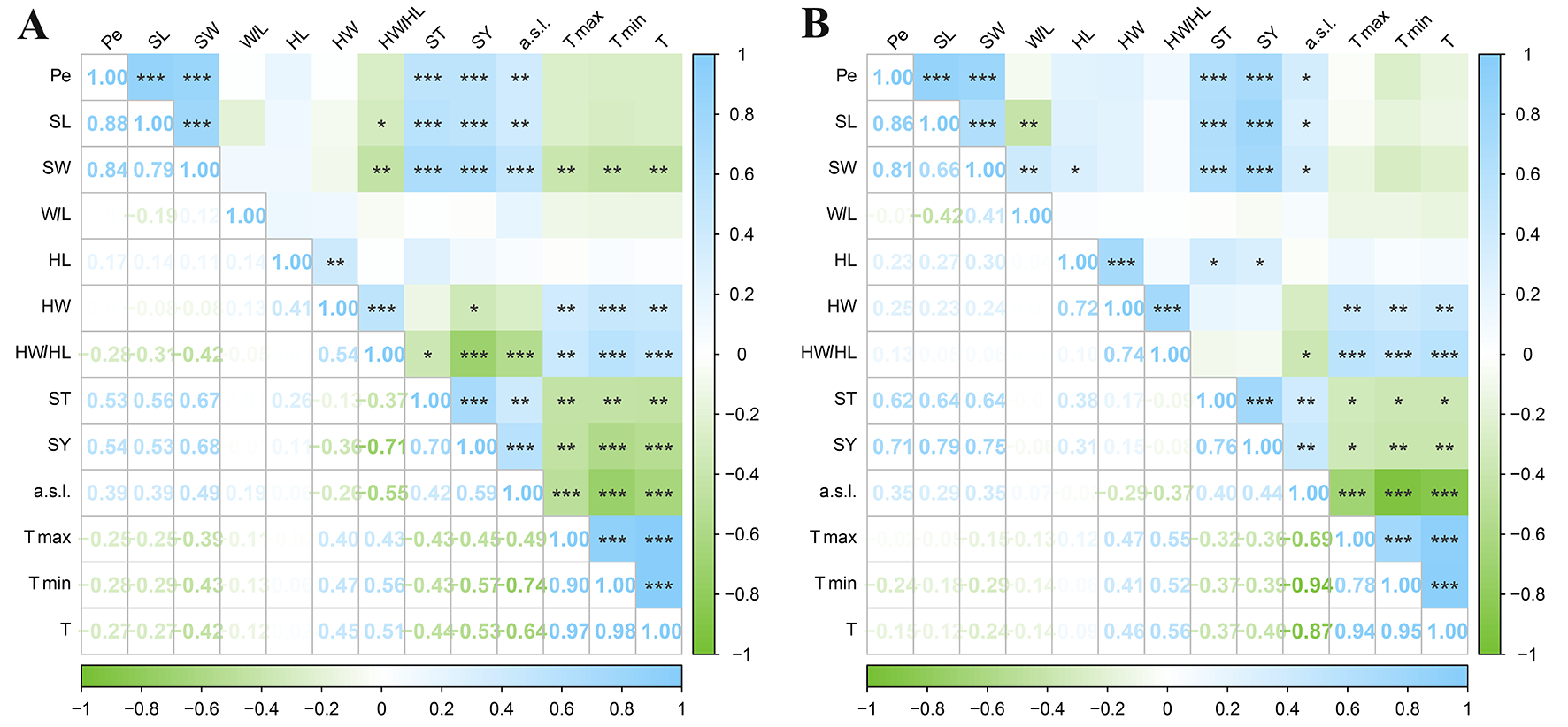

Supplement: Supplementary file 3 [file Image_2.tif]
